# Genes and regulatory mechanisms associated with experimentally-induced bovine respiratory disease identified using supervised machine learning methodology

**DOI:** 10.1038/s41598-021-02343-7

**Published:** 2021-11-25

**Authors:** Matthew A. Scott, Amelia R. Woolums, Cyprianna E. Swiderski, Andy D. Perkins, Bindu Nanduri

**Affiliations:** 1 Veterinary Education, Research, and Outreach Center, Texas A&M University and West Texas A&M University, Canyon, TX USA; 2 Department of Pathobiology and Population Medicine, Mississippi State University, Mississippi State, MS USA; 3 Department of Computer Science and Engineering, Mississippi State University, Mississippi State, MS USA; 4 Department of Comparative Biomedical Sciences, Mississippi State University, Mississippi State, MS USA

**Keywords:** Machine learning, Molecular medicine, Bioinformatics, Gene expression analysis, RNA sequencing, Infection, Respiratory tract diseases

## Abstract

Bovine respiratory disease (BRD) is a multifactorial disease involving complex host immune interactions shaped by pathogenic agents and environmental factors. Advancements in RNA sequencing and associated analytical methods are improving our understanding of host response related to BRD pathophysiology. Supervised machine learning (ML) approaches present one such method for analyzing new and previously published transcriptome data to identify novel disease-associated genes and mechanisms. Our objective was to apply ML models to lung and immunological tissue datasets acquired from previous clinical BRD experiments to identify genes that classify disease with high accuracy. Raw mRNA sequencing reads from 151 bovine datasets (n = 123 BRD, n = 28 control) were downloaded from NCBI-GEO. Quality filtered reads were assembled in a HISAT2/Stringtie2 pipeline. Raw gene counts for ML analysis were normalized, transformed, and analyzed with MLSeq, utilizing six ML models. Cross-validation parameters (fivefold, repeated 10 times) were applied to 70% of the compiled datasets for ML model training and parameter tuning; optimized ML models were tested with the remaining 30%. Downstream analysis of significant genes identified by the top ML models, based on classification accuracy for each etiological association, was performed within WebGestalt and Reactome (FDR ≤ 0.05). Nearest shrunken centroid and Poisson linear discriminant analysis with power transformation models identified 154 and 195 significant genes for IBR and BRSV, respectively; from these genes, the two ML models discriminated IBR and BRSV with 100% accuracy compared to sham controls. Significant genes classified by the top ML models in IBR (154) and BRSV (195), but not BVDV (74), were related to type I interferon production and IL-8 secretion, specifically in lymphoid tissue and not homogenized lung tissue. Genes identified in *Mannheimia haemolytica* infections (97) were involved in activating classical and alternative pathways of complement. Novel findings, including expression of genes related to reduced mitochondrial oxygenation and ATP synthesis in consolidated lung tissue, were discovered. Genes identified in each analysis represent distinct genomic events relevant to understanding and predicting clinical BRD. Our analysis demonstrates the utility of ML with published datasets for discovering functional information to support the prediction and understanding of clinical BRD.

## Introduction

Bovine respiratory disease (BRD) is the most important disease complex in beef cattle production. Although extensively researched, BRD remains the leading cause of infectious disease and economic loss in post-weaned beef cattle worldwide^1–4^. Due to the multifactorial and polymicrobial nature of BRD, effort has been made to illustrate host factors, management schema, etiological associations, and stressful environmental factors associated with disease development and progression^1,2,4^. Recent research has been focused on predicting BRD susceptibility and outcomes over time^5–8^. Unfortunately, clinical diagnostic and prognostic prediction models remain contested, and mechanistic information regarding host–pathogen interactions and the development of clinical BRD is not fully understood.

Clinical BRD is often linked with a select number of bacterial and viral etiologies. Bacteria, such as *Histophilus somni*, *Mannheimia haemolytica*, *Mycoplasma bovis*, and *Pasteurella multocida*, and viruses, such as bovine respiratory syncytial virus (BRSV), bovine viral diarrhea virus (BVDV), bovine herpesvirus-1 (IBR), and bovine parainfluenza type 3 virus (PI3), are well studied regarding their pathological capacity and disease association^9–15^. However, the clinical presentation of BRD is highly variable and antemortem diagnosis is often made without accompanying etiological identification^9,13,16,17^. Additionally, cattle experimentally exposed to these agents often fail to develop severe clinical BRD, demonstrating the underlying complexity of the disease and the requirement of implied predisposing factors^18,19^. Consequentially, current vaccination protocols possess varying effects in reducing ongoing rates of morbidity and mortality associated with BRD, and targeted antimicrobial usage and antimicrobial resistance is of particular public interest^20–25^. Therefore, research is needed to elucidate underlying host mechanisms associated with infectious BRD that represent biological components and regulatory functions amendable to manipulation to improve disease response and clinical diagnosis.

High-throughput RNA sequencing (RNA-Seq) is a highly sensitive methodology used to comprehensively evaluate functional mechanisms and molecular heterogeneity through global gene expression analysis^26–29^. Because of the high sensitivity of the technology, growing technological applications in research, and decreasing costs, RNA-Seq has become an excellent method of evaluating cellular transcriptomes and functionality at a given point in time. Several RNA-Seq studies performed with samples from post-weaned beef cattle have identified underlying genes and host mechanisms associated with both naturally occurring and experimentally induced BRD^30–35^. However, the results are highly dependent on the experimental design, sequencing technology, and selected data analysis technique, which may be highly conservative in nature^28,36–39^. Therefore, the use of supervised machine learning models with previously published RNA-Seq data could identify additional gene expression and mechanistic information related to clinical presentation of BRD.

Supervised machine learning (ML) models used in biological research aid in the discover of molecules and establishment of dynamic models that recognize, classify, and predict disease outcomes^40–44^. In recent years, studies have employed the use of ML framework to identify candidate biomarkers for disease classification, cell and tumor expression signatures, and novel protein mechanisms within publicly available RNA-Seq datasets^45–49^. However, to our knowledge, the use of ML-based methodology has not been explored with BRD-associated datasets. Therefore, we combined mRNA-Seq data from lung and immunological tissue of cattle experimentally challenged with causative agents of BRD, and tested the classification performance of ML methodology and selected gene classifiers. Our objective for this study was to integrate three publicly available datasets and utilize ML methodology, in order to both corroborate findings previously discovered through differential gene expression analysis and to potentially identify novel genes and mechanisms associated with experimentally induced BRD. Our overarching hypothesis is that ML methodology, when applied to previously published datasets, is capable of identifying genes which distinctly classify cattle challenged with etiological components of BRD, when compared to sham controls.

## Materials and methods

### Dataset acquisition

One hundred and sixty high throughput mRNA sequencing datasets were acquired from the National Center for Biotechnology Information (NCBI) Gene Expression Omnibus (GEO)^50,51^. The datasets originated from lymphoid and homogenized lung (healthy and diseased) tissue harvested during peak clinical signs in cattle that were experimentally challenged with isolated BRD pathogens (n = 35), or their sham controls (n = 10). Analyses of these datasets has been previously reported^30–32^. Details of sample sizes for challenged and control cattle, isolated BRD pathogens used for challenge, and tissue samples that were subjected to mRNA sequencing are summarized in Table 1.

**Table 1:** Initial training datasets identified for ML testing. A total of 160 mRNA-Seq datasets were derived from lymph node and lung tissue of 31 cattle challenged with isolated BRD pathogens and 10 sham challenged controls. Asterisk (*) indicates different tissues collected from the same animals. Specifically, transcriptomes from tissues reported by Behura et al.^31^ are from the same cattle from which Tizioto et al. analyzed bronchial lymph node transcriptomes (2015) except that *P. multocida* infected cattle reported by Tizioto et al.^30^ are not included in the cohort reported by Behura et al.^31^.

NCBI BioProject ID	Animal breed	Animal age	Number of animals	Tissue types	Etiological agents used in challenge	Sequencing platform	Publication
PRJNA272725	Angus × Hereford (steers)	6–8 mon	n = 23 challenge; n = 4 control	Bronchial lymph node	BRSV* (n = 4), BVDV* (n = 4), IBR* (n = 4), M. haemolytica* (n = 4), P. multocida* (n = 4), M. bovis* (n = 3), control* (n = 4)	Illumina HiSeq 2500; 50 bp PE	Tizioto et al., 2015 (n = 27)
PRJNA272725	Angus × Hereford (steers)	6–8 mon	n = 19 challenge; n = 4 control	Lung (healthy), lung (lesion), retropharyngeal lymph node, nasopharyngeal lymph node, pharyngeal tonsil	BRSV* (n = 4), BVDV* (n = 4), IBR* (n = 4), M. haemolytica* (n = 4), M. bovis* (n = 3), control* (n = 4)	Illumina HiSeq 2500; 50 bp PE	Behura et al., 2017 (n = 115)
PRJNA543752	Holstein–Friesian (bulls)	~ 4 mon	n = 12 challenge; n = 6 control	Bronchial lymph node	BRSV (n = 12), control (n = 6)	Illumina NextSeq 500; 75 bp PE	Johnston et al., 2019 (n = 18)

### Read processing and gene count matrix generation

Paired-end read files for each dataset were concatenated to their corresponding forward and reverse direction. To eliminate potential variations induced by differing workflow toolkits, all reads were processed identically. Quality assessment, read trimming, and adapter contamination removal was performed with FastQC v0.11.9^52^ and Trimmomatic v0.39^53^. Briefly, reads were trimmed by removing leading and trailing bases if base quality scores were less than 3, scanning each read with a 4-base pair sliding window and removing read segments below a minimum base quality score of 15, and retaining reads above a minimum length of 36 bases. Read quality analysis was summarized and evaluated for each study with MultiQC v0.37^54^. Read survival and quality assessment information are provided in Supplemental file 1. Trimmed reads were mapped to the bovine reference assembly ARS-UCD1.2 using HISAT2 v2.2.0^55^. Reference-guided transcript/gene assembly and quantification was performed with StringTie v2.1.2^56,57^. A gene-level raw count matrix was generated for each dataset with the program prepDE.py^58^. Five datasets [86684_Retrop_LN (control), 86688_Retrop_LN (BRSV), 86710_Retrop_LN (BVDV), 86698_dlung (*M. bovis*), and SRR1956908 (control)] were removed from further analysis due to low read count quantity and technical variability. Additionally, the four datasets related to *Pasteurella multocida* infection (SRR1952370, SRR1952371, SRR1952372, and SRR1952373) were removed to avoid unbalanced classification. The resulting compiled ML dataset was composed of 151 mRNA-Seq datasets.

### Supervised machine learning analysis

A total of 151 mRNA-Seq datasets, spanning six tissue types, constituted the compiled ML dataset for further classification and feature selection. Raw gene counts generated for each dataset were processed and analyzed in R v4.0.2 with the Bioconductor package MLSeq v2.6.0 (https://github.com/dncR/MLSeq)^59^. The 151 mRNA-Seq libraries were allocated into 9 classes based on the nature of the experimental pathogen challenge: (1) sham-challenged controls (Control; n = 28), (2) challenged with any BRD pathogen (BRD; n = 123), (3) challenged with a BRD viral pathogen (Virus; n = 82), (4) challenged with a BRD bacterial pathogen (Bacteria; n = 41), and categories 5–9 for each of the 5 independent challenge pathogens (BRSV; n = 35, BVDV; n = 23, IBR; n = 24, *M. haemolytica*; n = 24, and *M. bovis*; n = 17). The objectives of the ensuing ML analysis were to develop ML models that would (1) accurately “classify” an mRNA-Seq dataset within the 9 experimental pathogen challenge classes and (2) extract genes and gene sets or “features” that accurately assign an mRNA-Seq dataset to its experimental pathogen challenge class. These objectives were pursued by comparisons of the 8 pathogen challenge classes and the control challenge class. The raw gene count matrix used for this approach is available in Supplemental file 2. Briefly, offset values of one were added to the count matrix to reduce the likelihood of convergence in model fitting and to reduce bulk sparsity^60,61^. Genes with a minimum count-per-million of 0.5 in three or more mRNA-Seq libraries were retained for analysis. Library normalization was performed with the DESeq median ratio approach, using default settings^62^. The resulting ML dataset was stratified into a training and testing set (70% and 30%, respectively), using controls as the comparative baseline (i.e., class statement).

Model validation and parameter optimization were evaluated using fivefold, 10 repeats with non-exhaustive cross validation. Six ML models were utilized for classification and/or significant gene selection: sparse Poisson linear discriminant analysis, with and without a power transformation (PLDA, PLDA2)^63^, negative binomial linear discriminant analysis (NBLDA)^64^, sparse voom-based nearest shrunken centroids (VNSC)^65^, support vector machine (SVM) (https://cran.r-project.org/web/packages/caret/caret.pdf), and nearest shrunken centroids provided by the pamr package (PAM) (https://cran.r-project.org/web/packages/pamr/pamr.pdf). Models were evaluated with confusion matrices and performance metrics provided by the MLSeq package. Feature selection from sparse classifier models was set to a maximum of 2000 genes, based on maximum variance filtering. Sparse classifier models (PLDA, PLDA2, VNSC, and PAM), which generate lists of a select number of significant genes used for model decision and classification, were manually designated as the top models for each test set based on highest associated balanced accuracy and Kappa statistic; if two or more models were equal, gene lists would be merged. Performance metric calculations are defined by Goksuluk and colleagues^59^. Balanced accuracy, the combined average of sensitivity and specificity, was a prioritized metric due to imbalance between challenged and control cattle and potential for skewed results when evaluating sensitivity and specificity alone. Further information regarding workflow parameters, model building, and optimization are found in the package vignette and associated GitHub repository mirror (https://bioconductor.org/packages/release/bioc/html/MLSeq.html; https://github.com/dncR/MLSeq).

### Exploration and functional analysis of test set gene classifiers

Visual relationships of the genes identified by the top sparse classifiers was performed with UpSetR v1.4.0^66^, utilizing the interactive interface Intervene^67^. Multidimensional scaling was applied to the gene count matrix with plotMDS, using pairwise distances of the top 500 genes based on variance^68^. Heatmaps of the unique gene classifiers identified across etiologic test sets were generated with the Bioconductor package pheatmap v1.0.12^69^, utilizing Ward’s method of unsupervised hierarchical clustering on Euclidean distances and Pearson correlation coefficients for samples and genes, respectively. Color scaling for all packages was performed with the Bioconductor package viridis v0.5.1^70^ to allow ease of visual interpretation for individuals with color blindness.

Functional association and biological significance of genes from each test set was assessed. Gene Ontology (GO) terms and pathway analysis of DEGs was performed with WebGestalt 2019 (WEB-based GEne SeT AnaLysis Toolkit), utilizing human orthologs and functional databases^71^. Pathway analysis performed within WebGestalt 2019 utilized the pathway database Reactome^72^. Overrepresentation analysis parameters within WebGestalt 2019 included between 5 and 3000 genes per category, Benjamini–Hochberg procedure for multiple hypothesis correction, and FDR cutoff of 0.05 for significance.

## Results

### Supervised machine learning model performance

Mapping and alignment of reads to the ARS-UCD1.2 reference assembly identified 33,129 genes across all 151 libraries (n = 28 controls from 10 animals, n = 123 BRD from 32 animals; Supplemental file 2); the corresponding count matrix resulted in a total library size of 5,132,593,936, with a median library size of approximately 32.7 million counts per library. The count matrix was partitioned into nine pathogen challenge classes; overall testing performance for each ML algorithm is provided in Supplemental file 3. Support vectors machine (SVM) modeling, a non-sparse classifier, performed best in terms of balanced accuracy for all testing groups except for BVDV, which the nearest shrunken centroids model provided by the pamr package (PAM) outperformed all other models (86.7%). Because sparse classifiers select a subset of genes for classification^59^, genes were acquired and compiled from the top sparse models (PLDA, PLDA2, VNSC, or PAM) within each experimental challenge comparison. PAM performed best in terms of balanced accuracy when classifying Virus (89.9%), BRSV (100.0%), BVDV (86.7%), *M. bovis* (71.4%), and *M. haemolytica* (73.3%) against controls. Poisson linear discriminant analysis (PLDA) performed best when classifying Bacteria (70.0%). Both power-transformed Poisson linear discriminant analysis (PLDA2) and PAM performed identically when classifying IBR (100.0%). BVDV was less accurate (PAM; 86.7%), which most likely affected classification accuracy when evaluating all viral samples (PAM; 89.9%). Bacteria-challenged classes performed worse overall, with accompanying top balanced accuracies of 80.0%, 71.4%, and 80.0% for *M. haemolytica* (SVM), *M. bovis* (SVM/PAM), and Bacteria (SVM) classification, respectively. Combination of all challenge classes (BRD) possessed poor balanced classification accuracy, with the highest non-sparse classifier at 65.0% (SVM) and sparse classifiers (VNSC) at 60.8%.

### Visualization of gene expression variation

**Figure 1:**
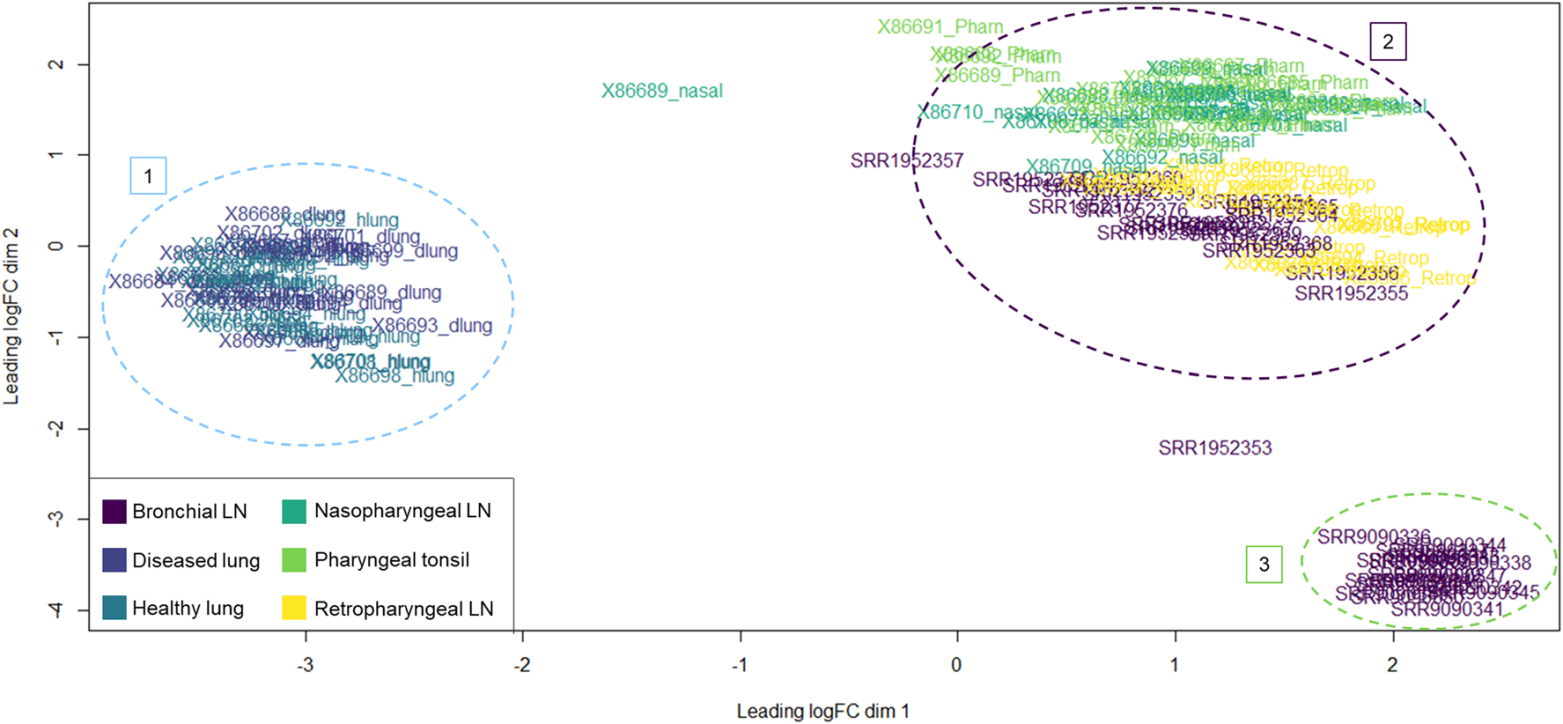
MDS plot of 151 datasets utilized for ML classification. Clustering was performed with Euclidian distances across the top 500 genes based on log2 standard deviation. Datasets are demarked by color, representing the tissue site of sampling. Labels 1, 2, and 3 demark distinct gene expression clusters across tissue types, regardless of etiological component, based on expressional variation. Label 1 consists of healthy (non-consolidated) and diseased (consolidated) lung tissue. Label 2 consists of lymphoid tissue from Tizioto and colleagues^30^ and Behura and colleagues^31^. Label 3 consists of lymphoid (bronchial) tissue from Johnston and colleagues^32^.

Multidimensional scaling (MDS) was applied to the integrated ML dataset, to discern dissimilarities between its individual mRNA-Seq libraries based on gene variation. Each point on x- and y-axes represents a different individual dataset and subsequent transformed Euclidean distance in two dimensions. Patterns from the top 500 genes based on log2-normalized standard deviation revealed that there was an overall similarity in gene expression across specific tissue types. While differences can be appreciated for each dataset with distinction to tissue site, lung (cluster 1; light blue) and lymphoid tissues (cluster 2; purple) were the most evident in terms of dissimilarity (Fig. 1). Notably, bronchial lymph node tissue from Johnston et al.^32^ (cluster 3; green) demonstrated equivalent dissimilarity from lung tissue as the bronchial lymph node tissue from Tizioto et al.^30^. However, the bronchial lymph node tissue from the two different studies were distinct from one-another when evaluated in the second dimensional space. Tissue-level gene expression differences (e.g., lung versus all other tissue types) were more pronounce compared to disease or etiological differences.

**Figure 2:**
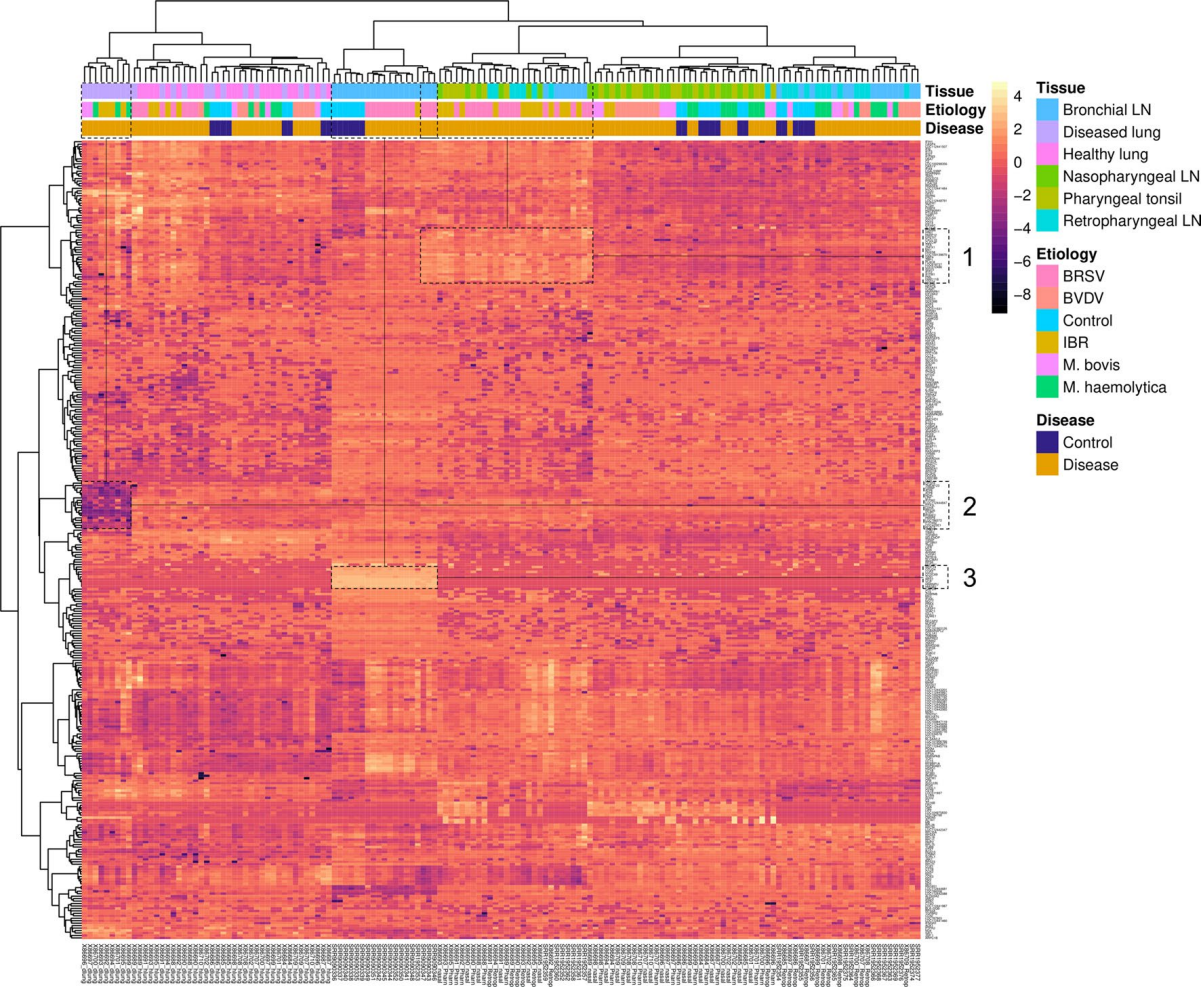
Heatmap of the 357 unique genes identified by top ML sparse classifier across the five etiology classes (BRSV, IBR, BVDV, *M. bovis*, and *M. haemolytica*). Ward clustering of datasets and gene expression was performed with Euclidian distance and Pearson correlation coefficients, respectively. Visual expression modules (1, 2 or 3) were empirically identified by class dissimilarity. Clustering of samples (datasets) is more apparent for tissue, compared to etiology and disease status.

A heat map was generated for each dataset using the gene classifiers identified through the top ML sparse model in each etiologic-specific test group (Fig. 2). A total of 572 genes were identified across the five etiological test groups, 357 of which were uniquely identified after overlapping (Supplemental file 4). Expression patterns within each column are accompanied by unsupervised hierarchical clustering, visualizing likeness in tissue type, etiology, and disease classification. Similar to the MDS plot (Fig. 1), distinction based on gene expression can be appreciated across lung and lymphoid tissue types, as opposed to etiology or disease classification. This distinction in gene expression across tissues corroborates the findings of Behura and colleagues^31^. Pearson correlation coefficients clustering of genes (rows) allowed for the visualization of distinct expression patterns. Particularly, three visual expression modules were identified, and labeled as 1, 2, and 3. Visual expression module 1 contained the genes *PSMB8, PPA1, PARP12, EPSTI1, CXCL10, CLEC4F, TIFA, ZNFX1, MX1, DHX58, LOC100139670, GBP4, ZBP1, PLAC8, LOC618737, LOC512486, ISG15, IFIT2, IFITM1, PML, FAM111B,* and *CD274*, which were distinctly higher in expression in lymphatic tissue sampled from cattle experimentally challenged with BRSV and IBR compared to all remaining. Visual expression module 2 contained the genes *CPSF6, TMEM123, CIRBP, ATP6, ATP8, ND4L, LPP, IFITM2, LOC112444847, DTX3L, LDHA, RPS26, STIP1, PSME2, PARP9, LOC786372, PTP4A2, CDC42SE1,* and *NLRC5*, which were distinctly decreased in disease lung tissue sampled from cattle experimentally challenged with *Mycoplasma bovis*, *Mannheimia haemolytica*, and IBR. Visual expression module 3 contained the genes *WDFY4, OTUD4, LCP2, OCDC69, TLN1, RPS7, VPC, HNRNPU,* and *HMGB2*, which were distinctly increased in bronchial lymph node tissue sampled from cattle in the control group and experimentally challenged BRSV.

**Figure 3:**
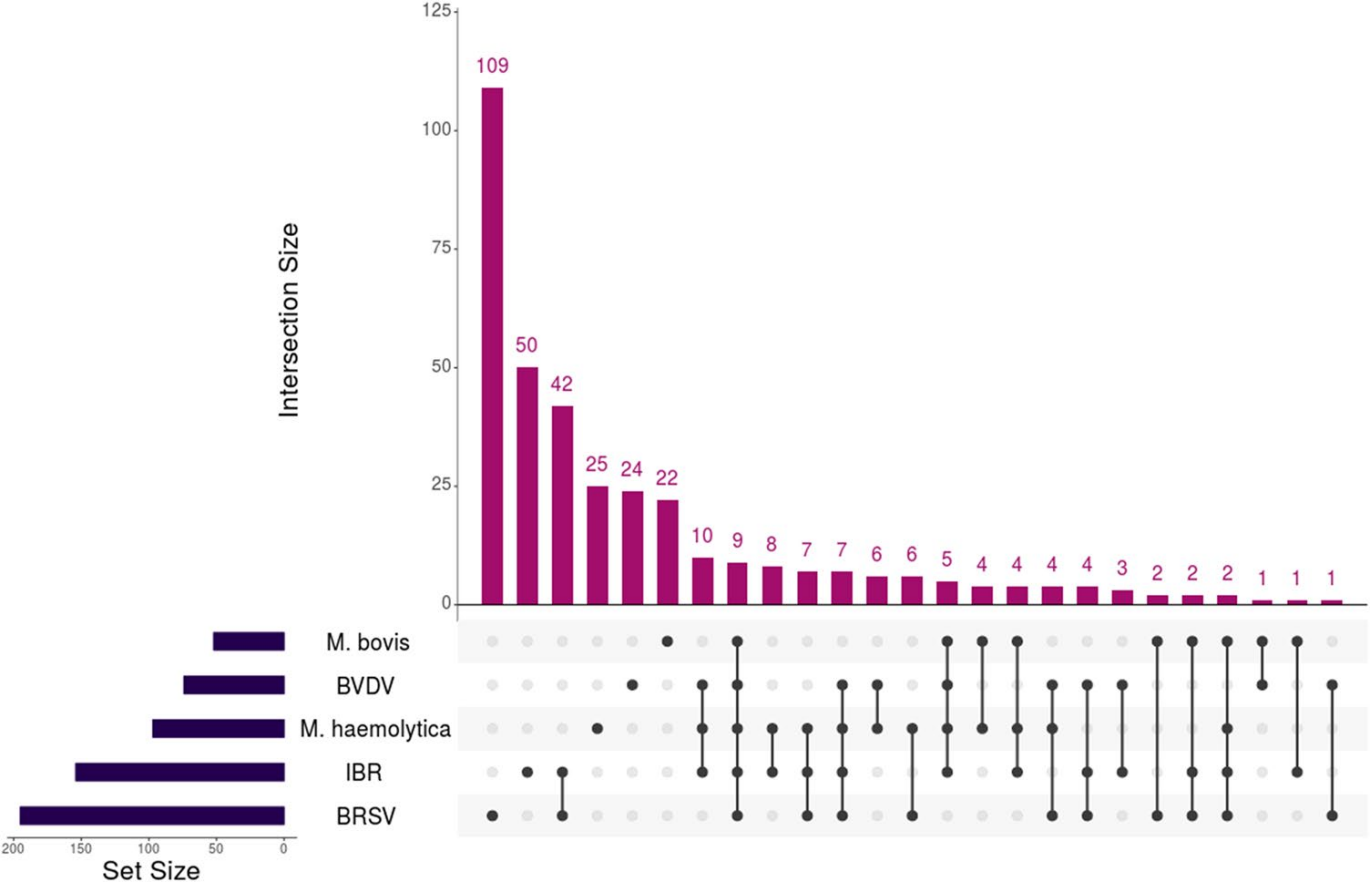
Matrix intersection of significant gene classifiers identified for each etiological class. Overlap of the 572 genes identified by top ML sparse classifiers, across the five etiology classes, were visualized for determining functional relevance and comparative uniqueness. BRSV possessed the highest number of uniquely identified genes (109), followed by IBR (50), *M. haemolytica* (25), BVDV (24), and *M. bovis* (22). BRSV and IBR shared the highest number of genes between all comparisons (42), primarily involved in type-I interferon production and signaling. The two bacterial classes (*M. haemolytica* and *M. bovis*) only shared four genes without any viral overlap (*LOC787803*, *MTDH*, *NECAP2*, and *TCAF1*).

To explore the complex overlap of gene classifiers between etiological groups, we employed an UpSetR matrix intersection technique (Fig. 3). Among the genes identified through the top sparse classifiers, BRSV was the most distinct with 109 unique genes. There was an apparent separation of viral-related genes, whereas BRSV and IBR possessed the highest overlap (42), BVDV possessed 24 unique genes, and only four genes were shared across all three viruses. Similarly, the bacterial datasets possessed minor overlap, with 25 and 22 genes identified uniquely for *M. haemolytica* and *M. bovis*, respectively, and only four genes shared between both bacterial analyses.

### Functional analysis of gene classifiers

**Figure 4:**
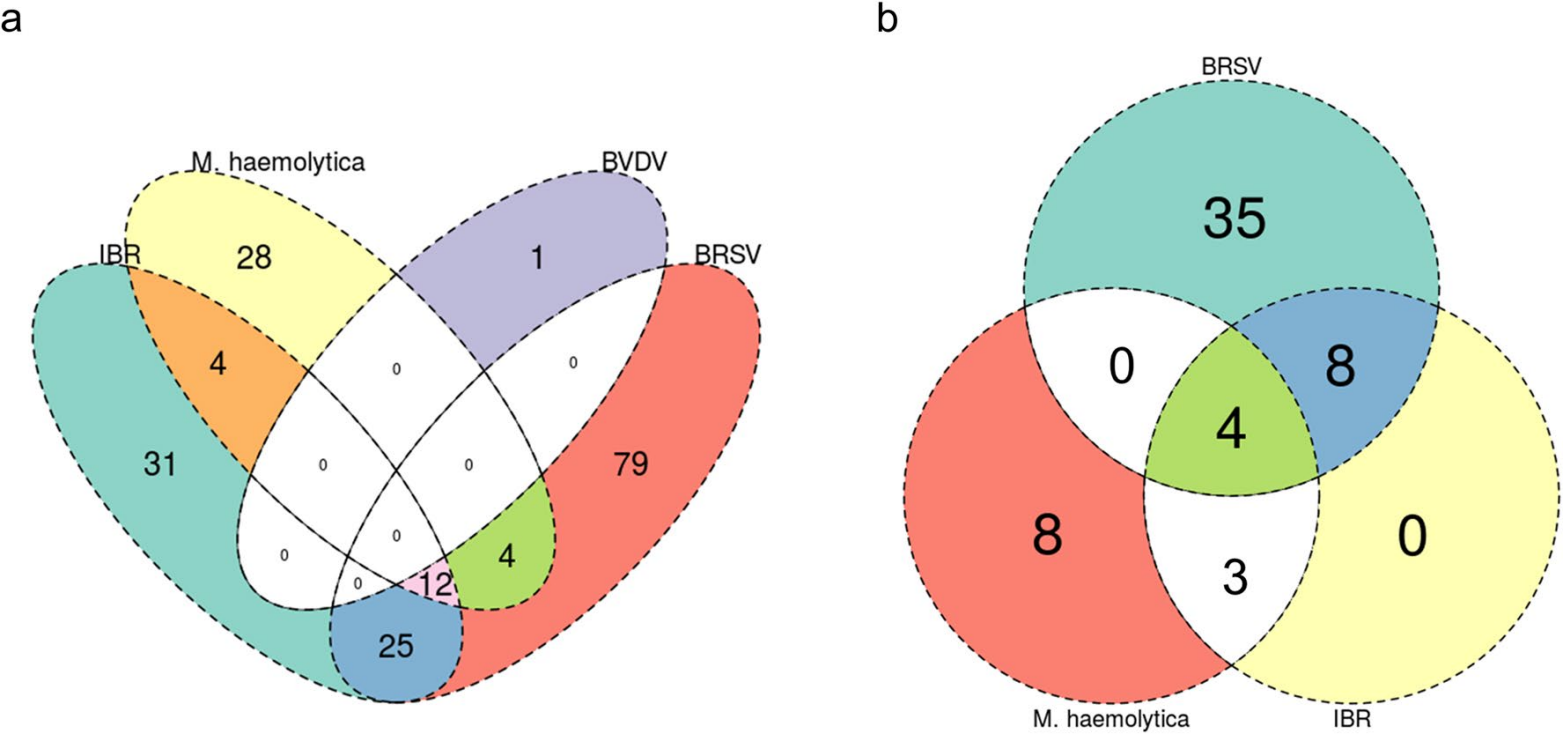
Venn diagram of GO-BP term (**a**) and pathways (**b**) enriched by genes identified by top ML sparse classifiers across all etiological testing sets. (**a**) Twenty-five enriched GO-BP terms were shared specifically for BRSV and IBR, primarily consisting of apoptotic processes, type 1 interferon signaling, IL-8 secretion, and leukocyte degranulation. BVDV possessed only one enriched GO-BP term (anatomical structure homeostasis) and no GO-BP terms were enriched for *M. bovis*. (**b**) Eight enriched pathways were shared specifically across BRSV and IBR, primarily consisting of antigen cross presentation, uptake of ligands by scavenger receptors, and interferon alpha/beta signaling. The four pathways shared across BRSV, IBR, and *M. haemolytica* involved the innate immune system, stress response element binding via ATF6-alpha, and signal recognition protein-dependent protein translation. The eight enriched pathways specific to *M. haemolytica* involved alternative complement activation, MHC class I antigen presentation, cellular response to heat stress, and IRE1-alpha-dependent chaperone activation. No pathways were enriched for BVDV or *M. bovis*.

Gene Ontology (GO) terms for biological processes and Reactome pathway enrichment analyses were performed with WebGestalt (FDR ≤ 0.05). One hundred and twenty, 72, one, and 48 GO-BP terms were significantly enriched by gene classifiers identified for BRSV, IBR, BVDV, and *M. haemolytica*, respectively; no significant GO-BP terms were enriched for *M. bovis*. Forty-seven, 15, and 15 pathways were enriched by gene classifiers identified for BRSV, IBR, and *M. haemolytica*, respectively; no pathways were enriched for BVDV and *M. bovis*. All GO-BP terms and pathways identified are found in Supplemental file 5. Overlap of the GO-BP terms and pathways identified for each etiological group is shown in Fig. 4A,B. BRSV and IBR possessed the highest overlap of functional associations, with 37 GO-BP terms and 12 pathways shared between the two. GO-BP terms and pathways between BRSV and IBR were primarily related to type I interferon production and signaling, cellular metabolism and ATP production, unfolded protein response, antigenic cross presentation, and IL-8 secretion. Between BRSV, IBR, and *M. haemolytica*, 12 GO-BP terms and 4 pathways were shared across all three. GO-BP terms and pathways between BRSV, IBR, and *M. haemolytica* were related to innate immune response, apoptosis, and unfolded protein response. *M. haemolytica* differed in functional enrichment with processes and pathways related to neutrophilic activation and degranulation, classical and alternative complement activation, and immunoglobulin-mediated humoral immunity. All five etiological groups shared genes involved in heat-shock protein response. The complete list of overlapping significant genes, GO-BP terms, and enriched pathways is found in Supplemental file 6.

## Discussion

Over the past several years, RNA-Seq analysis has been utilized in bovine disease research to evaluate gene expression related to risk of BRD development, stress response, and viral lesion development^30–35,73,74^. Primarily, studies that generate RNA-Seq data utilize statistical platforms and techniques for the detection of differentially expressed genes and subsequent construction of functional networks or modules. Many RNA-Seq studies are thus limited in extrapolatory capacity, as analyses are often performed through subsampling single populations and fitting fixed statistical models, which may be conservative when analyzing gene expression datasets with overdispersion^75–77^. Fortunately, publicly available gene expression repositories, such as the NCBI Gene Expression Omnibus, make it possible to acquire, integrate, and analyze datasets related to a particular field or disease. Such studies have been performed in mammalian species, including cattle, to better characterize genomic mechanisms and protein production related to a particular disease or condition^49,78,79^. Additionally, with the dynamic capacity for analysis that supervised ML models allow, it is possible to explore and characterize gene expression patterns associated with clinical BRD with profound sensitivity^42,79^. In this study, we integrated gene expression data from controlled BRD experiments and determine expression patterns and classification potential through supervised ML analysis.

Some of the limitations of this study are evident. First, data were integrated from three studies, two of which utilized the same animals for their transcriptomic analysis^30,31^. While a clear separation in gene expression patterns was appreciated across tissue types, corroborating the findings Behura and colleagues^31^, utilizing datasets from a limited number of animals and at single time points may not account for the heterogenous nature of gene expression related to BRD development and clinical presentation^75,80^. Additionally, these datasets were acquired from samples of cattle experimentally challenged with single pathogens. BRD challenge models using single etiological components often fail to elicit severe disease, as described by the three studies used here and may not fully represent the complex nature of the disease process seen with naturally occurring BRD^81,82^. Accordingly, future studies applying ML methodology in BRD research should prioritize natural disease models for improved discovery adaptation within beef production systems. Moreover, RNA-Seq analysis remains a relatively new modality in BRD research, and publicly available data are limited at this time. Nonetheless, this study, which to our knowledge is the first to evaluate host transcriptomes related to BRD with integrated supervised ML methodology, substantiating many of the gene expression findings previously identified, and may serve as a template for modern data analysis in bovine health research.

Between all testing groups and the six models utilized in this study, the support vector machines (SVM) model typically performed the best in terms of classification capacity. While originally utilized in microarray experiments, this algorithm is popular for genomic classification research in RNA-Seq, as it has been used to discover cancer biomarkers in humans, classify genes related to early conception in cattle, and automate single-cell RNA-Seq identification^49,83,84^. While this algorithm was capable of accurately classifying BRSV and IBR challenged datasets, compared to controls, this model is a non-sparse classifier and therefore does not have a built-in process for feature selection and gene extraction within MLSeq. Therefore, particular interest was placed on the PLDA, PLDA2, PAM, and VNSC algorithms, as subsets of genes used to drive classification decisions could be obtained. The compiling of datasets for classifying total BRD, viral, and bacterial challenge yielded mixed results. For total BRD, sparse classifiers PAM and VNSC yielded high classificational accuracy for identifying the challenged cattle, but performed poorly in discerning them from the controls, as illustrated by the accompanying sensitivity and balanced accuracy. This finding may be related to the complexity and distinction of infection processes associated with each etiological component, and highlights that an all-encompassing approach may be inappropriate for determining relevant gene expression and pathways in BRD. To a lesser extent, this is similarly found when compiling bacterial datasets, as discernment from controls was relatively poor. Viral datasets yielded much higher overall balanced accuracies, compared to the bacterial counterparts. Regarding sparse classifiers, BRSV, IBR, and BVDV were capable of being classified with high balanced accuracy (100%, 100%, 86.67%, respectively) through the PAM model; IBR was also identified with 100% balanced accuracy with PLDA2.

Generally, viruses were independently the most well classified, followed by *M. haemolytica*. Collectively, BRSV and IBR were well defined by genes involved in the production and response to type I interferons. More specifically, these genes were seen to be driven primarily by lymphoid tissue, as opposed to lung tissue (expression module 1, Fig. 2). This result, coupled with the subsequent lack of type I interferon genes from the BVDV class, corroborates findings previously described^30,31^. Biologically, the lack of this innate interferon response has been described as a potential immunosuppressive response driven by BVDV, allowing for persistent infection and viral shedding^85–88^. Notably, non-cytopathic BVDV strains used in experimental infection models, such as the one utilized in this project, have been shown to reduce proinflammatory signaling^31,89^. While the functional enrichment of the genes classified for BVDV were largely non-specific, several have been previously identified and have known immunological functionality^30,31^. Related to *M. haemolytica*, there was apparent overlap in functionality of genes identified through ML (Fig. 4). Largely, this was driven by genes encoding for heat shock proteins, calreticulin, talin-1, and X-box binding protein. These proteins are involved in apoptotic and cell stress events, and may ultimately impact immunoglobulin production and cellular homeostasis^90–93^. Additionally, genes classified in *M. haemolytica* were unique to the activation of classical and alternative pathways of complement. While complement-related genes were identified across all viruses in previously reported gene expression studies and here, the alternative complement component *CFB* was only identified in *M. haemolytica*. This may be an indication that the presence and activation of the alternative complement pathway is more indicative of inflammation and disease associated with lipopolysaccharide, often caused by extracellular Gram-negative bacteria such as *M. haemolytica*^14,94^. Regarding *Mycoplasma bovis*, our findings here are similar to that of Behura and colleagues^31^, in that we identified the fewest number of significant genes in MB, with regard to all other classes, and failed to define significantly enriched processes and pathways. As discussed by Behura and colleagues^31^, these infected cattle may have been euthanized and sample collected too early in the course of BRD to detect immunological changes. Additionally, *Mycoplasma bovis* is capable of evading the host immune response, specifically neutrophilic responses, and may lead to the development of T-cell “exhaustion” that eventually culminates in severe clinical disease^95^. Future transcriptomic evaluation of single cells or the sub-cellular space, instead of bulk tissue samples, may better elucidate mechanisms associated with *Mycoplasma bovis*.

Lastly, novel findings were identified through visual expression modules found in Fig. 2. Expression module 2 possessed 19 genes with reduced expression in disease lung tissue sampled from cattle experimentally challenged with *Mycoplasma bovis*, *Mannheimia haemolytica*, and IBR. While often assumed that the oxygenating capability of consolidated lung space during acute respiratory disease is substantially decreased, this expression module provides evidence of this event, as these genes largely possess aerobic ATP synthase and mitochondrial function^96–99^. Expression module 3 had nine genes with increased expression in bronchial lymph node tissue sampled from cattle in the control group and BRSV. These genes play important roles in T-cell proliferation, integrin activation and antigenic presentation through actin/tubulin reorganization^100–103^. Potentially, this serves as an underlying mechanism of immunological stimulation unique to lymph nodes of the lower airway.

## Conclusion

This study was conducted to integrate and analyze mRNA-Seq datasets with supervised ML methodology. This approach allowed for a novel and comprehensive analysis of lung and immunological tissues in to experimentally induced BRD. ML enabled the classification of viral-induced BRD, specifically with BRSV and IBR, with 100% balanced accuracy, against sham controls, regardless of the tissue type. This experimental investigation illustrates a novel and powerful approach to the investigation of host response mechanisms in BRD through the use of mRNA-Seq and supervised ML analysis.

## Supplementary Information

Supplementary Information 1.

Supplementary Information 2.

Supplementary Information 3.

Supplementary Information 4.

Supplementary Information 5.

Supplementary Information 6.

## Data Availability

The data utilized in this study were downloaded from the National Center for Biotechnology Information Gene Expression Omnibus (NCBI-GEO), Bioproject numbers PRJNA272725 and PRJNA543752.
